# Effects of Raffinose on Growth Performance, Intestinal Function-Related Genes, and Cecal Microbiota in Broilers Fed Low Soybean Meal Diets

**DOI:** 10.3390/ani16060928

**Published:** 2026-03-16

**Authors:** Xiang Lan, Shiping Bai, Gang Tian, Gang Lv, Keying Zhang, Jiang Yuan, Xuemei Ding, Jianping Wang, Yan Liu, Yue Xuan, Shanshan Li, Qiufeng Zeng

**Affiliations:** 1 Key Laboratory of Animal Disease-Resistance Nutrition Ministry of Education, Key Laboratory of Sichuan Province, Animal Nutrition Institute, Ministry of Agriculture and Rural Affairs, Sichuan Agricultural University, Chengdu 611130, China; 2023214011@stu.sicau.edu.cn (X.L.); shipingbai@sicau.edu.cn (S.B.); 13555@sicau.edu.cn (G.T.); zkeying@sicau.edu.cn (K.Z.); dingxuemei0306@163.com (X.D.); wangjianping@sicau.edu.cn (J.W.); liuyan12334@163.com (Y.L.); 71128@sicau.edu.cn (Y.X.); 71449@sicau.edu.cn (S.L.); 2 Xinjiang Taikun Group Co., Ltd., Changji 831100, China; lvgang@feederbio.com (G.L.); 18999361205@189.cn (J.Y.)

**Keywords:** broiler, gut microbiota, low soybean meal, nutrient utilization, raffinose

## Abstract

This study investigated the effects of a low soybean meal (SBM) diet and its supplementation with graded levels of raffinose on the growth performance, expression of genes related to nutrient transport and intestinal function, and cecal microbiota of white-feathered broilers. A total of 480 one-day-old Cobb broilers were randomly allotted to six isoenergetic and isonitrogenous dietary treatments, each with eight replicates of 10 birds. The diets consisted of a positive diet, a low SBM diet (10% reduction in SBM), and the low SBM diet supplemented with 0.10%, 0.15%, 0.20%, or 0.25% raffinose. Results indicated that, compared with the positive diet, the low SBM diet significantly increased (*p* < 0.05) the overall mortality and average daily feed intake (ADFI) during days 22–42, while significantly decreasing (*p* < 0.05) dietary ether extract (EE) availability. Raffinose supplementation to the low SBM diet linearly reduced (*p* < 0.05) dietary gross energy and dry matter utilization and downregulated duodenal SLC5A1 gene expression at 42 days, while linearly increasing (*p* < 0.05) the cecal isobutyric acid content. A decreasing tendency in mortality during days 22–42 was also observed with raffinose inclusion (*p* = 0.088). Notably, the low SBM diet elevated the relative abundance of Campylobacterota and *Helicobacter*, which was effectively reversed by raffinose supplementation. In conclusion, a 10% reduction in dietary SBM negatively affected the survival, nutrient utilization, and cecal microbial structure in broilers, whereas raffinose supplementation partially modulated these alterations.

## Simple Summary

The modern broiler industry is crucial for supplying high-quality animal protein globally, yet this industry requires a large amount of expensive and resource scarce soybean meal (SBM) as a source of feed protein. Reducing the amount of SBM in broiler feed is a great challenge. In this context, we first used a low SBM diet to investigate the effects of functional oligosaccharides (raffinose) in SBM on the productive performance, intestinal health, and gut microbiota in broiler chickens. Raffinose can pass intact into the hindgut, acting as a specific nutrient for beneficial bacteria such as *Bifidobacterium* and *Lactobacillus*. The results showed that low SBM diet application in white-feathered broiler diets need to consider the content of functional soybean oligosaccharide, which provide a new perspective for reducing SBM consumption in the poultry feed industry.

## 1. Introduction

Soybean meal (SBM) is the dominant and widely used protein source in poultry nutrition due to its high protein content and superior amino acid [1]. However, the reliance on SBM in poultry diets raises concerns regarding economic volatility, environmental footprint, and social sustainability, particularly in arid regions and import-dependent countries [2,3]. Consequently, there is an urgent need to reduce SBM inclusion in broiler feed. However, the partial replacement of SBM with unconventional protein sources (e.g., cottonseed meal, rapeseed meal) often yields suboptimal results [4]. The reason may be related to the presence of special functional substances in SBM.

Soybean carbohydrates account for approximately 40% of soybean meal dry matter [5]. Previous studies have showed that soybean oligosaccharide reduces growth performance, nutrient digestibility and causes flatulence symptom in pigs [6,7]. Thus, traditional nutrition indicates that soybean oligosaccharides, such as raffinose and stachyose, are antinutritional factors. However, recently, the use of bioactive compounds, such as prebiotics, has been shown to be very attractive, given their role in modulating the gut microbiota and the subsequent beneficial effects on human and animal health over different physiological functions. Xu et al. [8] found that adding a moderate amount of soybean oligosaccharides to basic diets helped white-feathered broilers to use protein, phosphorus, and calcium better. Among soybean oligosaccharides, raffinose, a trisaccharide composed of galactose, glucose, and fructose (also known as melitriose), accounts for approximately 12.6 mg/g of dry soybean seed [9,10]. This trisaccharide is non-digestible to humans and animals or poultry but can be metabolized by colon bacteria [11], and is therefore considered a prebiotic candidate. Many previous studies showed that raffinose helps good bacteria grow, keeps bad bacteria from multiplying, and supports the immune system, fights oxidation, and protects the liver, which helps to maintain a healthy balance of gut bacteria in animals [12,13]. Shehata et al. [14] observed that raffinose promotes daily weight gain in broilers while inhibiting the proliferation of *Escherichia coli* and *Salmonella*, improves nutrient digestibility, such as crude protein and crude fat, in weaned piglets [15], and improves gut microbiota abundance, thereby augmenting host health and nutrient utilization through prebiotic–microbe interactions [16]. In yellow-feathered broilers, Yushanaji et al. [17] found that adding 0.1% raffinose to their diet helped their growth, gut bacteria, and health.

Based on the above studies, we hypothesized that oligosaccharides present in SBM might be one of the key factors limiting the application of low SBM diets in the poultry industry. Up to now, studies investigating the supplementation of oligosaccharides in a low SBM diet are scarce. Therefore, this study aimed to clarify the effects and appropriate amounts of supplementing graded levels of raffinose into the low SBM diet (with a 10% reduction) on growth performance, nutrient utilization, intestinal nutrient transport- and function-related gene expression, cecal microbiota, and microbial metabolites in white-feathered broilers, providing a theoretical basis for reducing soybean substitution in the poultry feed industry.

## 2. Materials and Methods

### 2.1. Birds, Experimental Design, Diet, and Management

A total of 480 one-day-old healthy Cobb broilers were randomly allocated into 6 treatment groups, with 8 replicates per treatment and 10 chicks per replicate. The chickens were obtained from Zhengda Food Suining Co., Ltd. (Suining, China). Six isocaloric and isonitrogenous experimental diets were formulated: a positive diet, a low SBM diet (with a 10% reduction in SBM), and the low SBM diet supplemented with 0.10%, 0.15%, 0.20%, or 0.25% raffinose (purity > 94.0%, SinoLeader Biotech, Beijing, China). The composition and nutritional levels of the positive diet and the low SBM diet were as presented in Table 1. All diets were provided in pellet form.

The experiment lasted for 42 days, divided into a starter phase (days 1–21) and a finisher phase (days 22–42). Broilers were housed in cages (1.0 × 0.8 × 0.6 m) with ten birds per cage. The room temperature was maintained at 34 °C for the first three days and then gradually reduced by 2–3 °C weekly until reaching a final temperature of 22 °C. The relative humidity was controlled at 50–60%. All broilers had ad libitum access to feed and water.

### 2.2. Data and Sample Collection

On days 21 and 42, after a 12 h fast, birds were weighed individually, and feed intake per cage was recorded. Body weight (BW), average daily gain (ADG), average daily feed intake (ADFI), feed-to-gain ratio (F:G) and mortality rate were calculated for the starter, finisher, and overall periods, where the F:G is adjusted using the mortality rate. The European Performance Index (EPI) is calculated as follows: EPI = [Survival Rate × Live Weight (kg)]/(F:G × Days to Market) × 10,000.

On days 21 and 42, one bird per replicate with a body weight close to the replicate average was selected for sampling. Mucosa samples from the duodenum, jejunum, and ileum, as well as digesta from the cecum, were collected and immediately stored at −80 °C for subsequent gene expression and microbial analysis.

### 2.3. Assay of Nutrient Utilization

On day 43, two broilers from each replicate were transferred to metabolic cages and fed their original diets supplemented with 0.5% titanium dioxide (TiO_2_) as an inert marker. Excreta samples were collected from each cage over a period of 72 h. Following the removal of any debris, the samples were gathered in each cage and dried in an oven at 65 °C for 3 days. All samples were ground to pass through a 0.5 mm screen and then analyzed for dry matter (DM), gross energy (GE), ether extract (EE), nitrogen (N), calcium (Ca), and total phosphorus (TP), in accordance with the method (AOAC, 2005) [18]. Crude protein (CP) was calculated as N × 6.25. EE in the diets and excreta was measured with a Soxhlet apparatus for approximately 8 h. GE was analyzed by using a Parr 6400 oxygen bomb calorimeter (Parr Instrument Co., Moline, IL, USA). The TiO_2_ content in feed and excreta samples was measured according to the method proposed by Short et al. [19]. Nutrient utilization for the experimental diets was calculated with the following formula: nutrient utilization (%) = {1 − [(N_e_ × T_d_)/(N_d_ × T_e_)]} × 100, where T_e_ = TiO_2_ concentration in excreta (% dry matter, DM), T_d_ = TiO_2_ concentration in the diet (% DM), N_e_ = nutrient concentration in excreta (% DM), and N_d_ = nutrient concentration in the diet (% DM).

### 2.4. Gene Expression Assays

Total RNA was extracted from frozen duodenal, jejunal and ileal mucosa samples using a Trizol reagent (TaKaRa, Dalian, China), and first-strand cDNA synthesis was performed with the PrimeScript™ RT Reagent Kit (Takara, Dalian, China), in accordance with the manufacturer’s instructions. The quantitative real-time PCR (qRT-PCR) was performed on the ABI QuantStudio™ 6 Flex system (Applied Biosystems, Waltham, MA, USA). The primer sequences for the target genes were designed using the National Centre for Biotechnology Information (NCBI) Blast tool. All primers set in the qRT-PCR reaction were run for melting curve analyses to generate a standard curve to assess PCR efficiency. A comprehensive list of all primer sequences used in this study is presented in Table 2. Relative gene expression was quantified by normalizing to the expression of β-actin according to the 2^−ΔΔCt^ method, with the quantity of the positive group scaled to approximately 1.

### 2.5. Detection of Short Chain Fatty Acids in the Digesta of Cecum

Concentrations of main short chain fatty acids (SCFA, including acetate, propionate, butyrate, isobutyrate and branched chain valerate) in the cecal digesta samples were determined by gas chromatography (GC CP3800, Varian, Palo Alto, CA, USA). After thawing, about 0.5 g of the cecal digesta was evenly sampled and 1.2 mL of the ultrapure water was added, homogenized, and centrifuged at 14,000 rpm for 15 min after standing for 5 min. We added 0.2 mL of 25% (*w*/*v*) metaphosphoric acid solution and 23.3 μL of 210 mmol/L crotonic acid solution to 1 mL of supernatant, mixed well, and then incubated it for 30 min at 4 degrees, and centrifuged it at 8000 rpm for 10 min. After centrifugation, we took 0.3 mL of supernatant and added 0.9 mL of chromatographic methanol to mix well. We centrifuged it at 8000 rpm for 5 min. We took the supernatant and filtered it with 0.22 μM filter membrane and then used the machine for determination.

### 2.6. 16S rRNA Gene Sequencing

The raw sequencing data were processed as follows. First, quality filtering was performed using Trimmomatic [20] (version 0.33). Primer sequences were then identified and removed using Cutadapt [21] (version 1.8.3). The subsequent steps differed, based on the chosen clustering method. For the denoising (dada2) method, we used the dada2 package in R for further quality control, merging of paired-end reads, and chimera removal. For the similarity-based clustering method, we used USEARCH [22] (version 10) to merge paired-end reads and remove chimeras using UCHIME [23] (version 8.1), resulting in high-quality sequences for downstream analysis. The specific parameters were as follows: (1) Trimmomatic quality control: Trimmomatic is a quality control tool for filtering Illumina high-throughput sequencing reads. It processes FASTQ format data (both paired-end and single-end) with base quality scores in either a phred 33 or phred 64 format (depending on the Illumina sequencing platform). Single-end data require one input and one output file name plus parameters, while paired-end data require two input files (forward and reverse FASTQ data). The parameters were set as follows: a sliding window of 50 bp; if the average quality within the window fell below 20, the trailing bases from the start of the window were trimmed off. (2) Primer identification and removal: the Cutadapt software (version 2.7) was used with parameters set to allow a maximum mismatch rate of 20% and a minimum overlap of 15 bp for primer identification. Subsequent length filtering was applied based on the targeted amplicon region. For the common 16S V3–V4 region, a length threshold of 350 bp to 490 bp was used. (3) dada2 processing: the filterAndTrim function was used for further quality control, with maxEE set to 2 (where EE = sum(10^(−Q/10))) and other parameters at the default. The error model was built using the learnErrors function. Denoising was performed with the dada function. Paired-end reads were merged, using the mergePairs function with parameters: minOverlap = 18 and maxMismatch = 18 × 0.2. Chimeras were removed using the removeBimeraDenovo function (choosing the “consensus” method). (4) Paired-end read merging and chimera removal (for similarity clustering): the USEARCH v10 software was used to merge reads for each sample with the following parameters: a minimum overlap length of 10 bp, a minimum overlap identity of 90%, and a maximum of 5 mismatched bases allowed in the overlap region (default). Chimeras were subsequently removed using UCHIME [23] (version 8.1) within USEARCH [22] (version 10).

### 2.7. Statistical Analysis

Data were analyzed using SAS software 9.4 (SAS Institute Inc., Cary, NC, USA). Data from the positive diet and the low SBM diet groups were compared using Student’s *t*-test. Data from the low SBM diet and the raffinose-supplemented groups were subjected to one-way analysis of variance (ANOVA), followed by Tukey’s multiple range test. The general linear model procedure was conducted for linear and quadratic analyses. Differences were considered significant at *p* < 0.05, and trends were discussed at 0.05 < *p* < 0.10. Data are presented as the mean and standard error of the mean (SEM).

## 3. Results

### 3.1. Growth Performance

As shown in Table 3, the low SBM diet had no significant effect (*p* > 0.05) on growth performance parameters compared to the PC diet, except for a significant increase (*p* < 0.05) in ADFI during days 22–42 and in overall mortality (days 1–42). Supplementation of raffinose to the low SBM diet did not significantly affect ADG, ADFI, or F:G (*p* > 0.05). However, raffinose supplementation had a significant effect on the mortality of broilers (F = 2.66, *p* = 0.049); the post comparison displayed mortality during days 1–21 was significantly lower (*p* < 0.05) in the 0.25% raffinose group compared with the low SBM group. Furthermore, a linear decreasing tendency (*p* = 0.088) in mortality during days 22–42 was observed with increasing raffinose levels.

### 3.2. Nutrient Utilization

Dietary EE availability was significantly higher (*p* < 0.05) in the positive group than in the low SBM group (Table 4), and raffinose supplementation had a significant effect on the DM availability of broilers (F = 2.90, *p* = 0.036), the post comparison displayed that DM availability was significantly higher (*p* < 0.05) in the 0.15% raffinose group compared with the 0.25% group. In broilers fed the low SBM diet, raffinose supplementation linearly decreased (*p* < 0.05) the utilization of DM and GE, and quadratically affected (*p* < 0.05) the utilization of CP and TP.

### 3.3. Gene Expression

#### 3.3.1. Nutrient Transport-Related Genes

The low SBM diet had no significant effect (*p* > 0.05) on the mRNA expression of duodenal and jejunal glucose transporters (SLC5A1 and SLC2A2) at either 21 or 42 days of age (Table 5). However, raffinose supplementation had a significant effect on the expression of SLC5A1 on day 42 in the broilers’ duodenum (F = 2.72, *p* = 0.046); the post comparison displayed that the gene expression was significantly lower (*p* < 0.05) in the 0.15% and 0.20% raffinose groups compared to the low SBM group. Raffinose supplementation linearly downregulated (*p* < 0.05) the expression of duodenal SLC5A1 at 42 days, while linearly upregulating (*p* < 0.05) jejunal SLC5A1 expression at the same age.

#### 3.3.2. Ileal Barrier Function-Related Genes

No significant differences (*p* > 0.05) were observed in the expression of ileal tight junction-related genes (OCLN, TJP1, CLDN1, CLDN3, MUC2) between the positive diet and low SBM groups (Table 6). Within the raffinose-supplemented groups, the expression of CLDN1 at 42 days showed a quadratic response (*p* < 0.05), and a similar trend was observed for TJP1 (*p* = 0.093).

### 3.4. Cecal Short-Chain Fatty Acid Content

With the exception of isobutyric acid, no significant differences (*p* > 0.05) in cecal SCFA concentrations were found among groups (Table 7). Raffinose supplementation had a significant effect on the isobutyric acid concentrations in broilers (F = 23.76, *p* < 0.001); the post comparison displayed raffinose supplementation at 0.20% and 0.25% as significantly increasing (*p* < 0.05) the concentration of isobutyric acid compared to the low SBM diet. A linear and quadratic increase (*p* < 0.05) in the isobutyric acid content was observed with increasing raffinose levels.

### 3.5. Cecal Microbiota

#### 3.5.1. Cecal Microbial Alpha Diversity

**Figure 1:**
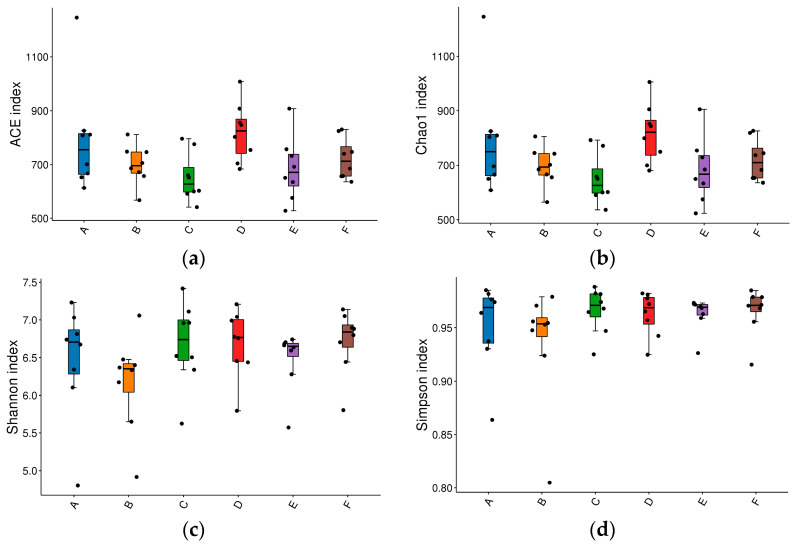
The effects of raffinose on the cecum microbiota in broilers: (**a**) ACE index; (**b**) Chao1 index; (**c**) Shannon index and (**d**) Simpson index. A, B, C, D, E and F are the positive diet, low SBM diet, low SBM with 0.10%, 0.15%, 0.20% and 0.25% raffinose supplementation groups, respectively. Individual data points represent each sample. Different colors indicate different groups (A to F).

The microbial complexity in the cecum was estimated on the basis of alpha-diversity indices (Chao1 index, ACE index, Simpson index and Shannon index). The Chao1 index and ACE index were used to estimate species richness, and the indexes of Shannon and Simpson were used to indicate species diversity. There was no difference in the ACE, Chao1, Shannon and Simpson indices among groups, although the ACE and Chao1 index in the 0.15% group was the highest, while the Shannon index in the low SBM diet group was the lowest (*p* > 0.05) (Figure 1).

#### 3.5.2. Cecal Microbial Relative Abundance Analysis

**Figure 2:**
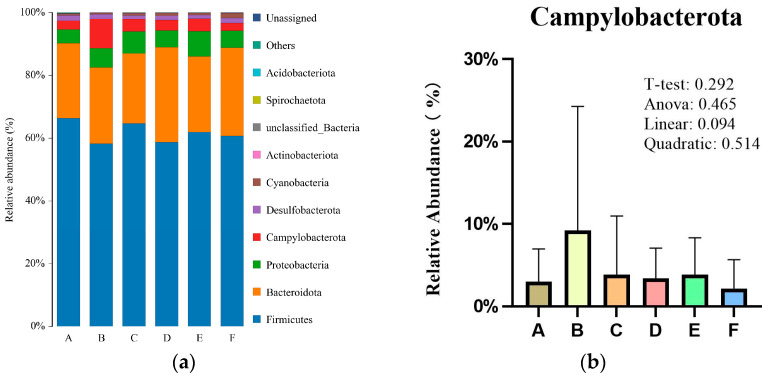
A, B, C, D, E and F are the positive diet, low SBM diet and low SBM with 0.10%, 0.15%, 0.20% and 0.25% raffinose supplementation groups, respectively. (**a**) Relative abundance of bacterial phylum in the cecal digesta of broilers; (**b**) Relative abundance of Campylobacterota at the phylum level. All colors represent Campylobacterota. The *t*-test is the *p*-value for the comparison of the positive diet versus the low SBM group, while the Anova, linear, and quadratic are the *p*-values for the comparisons among the low SBM and low SBM with 0.10%, 0.15%, 0.20% and 0.25% raffinose supplementation groups, respectively.

There were six phyla with proportions greater than 0.1% (Figure 2a): Firmicutes, Bacteroidetes, Proteobacteria, Campylobacterota, Desulfobacterota, and Cyanobacteria. Firmicutes was the dominant phylum in the cecal microbiota, accounting for 61% of the overall bacteria community, although no significant difference was observed among groups. Bacteroidetes was the second most abundant (24%) phylum in the cecum, and the average abundance value of both Firmicutes and Bacteroidetes was over 94.6% in all groups. The relative abundance of Campylobacterota (9.22%) was highest in the low SBM diet group and showed a linear decreasing trend (*p* = 0.094) with raffinose supplementation (Figure 2b).

**Figure 3:**
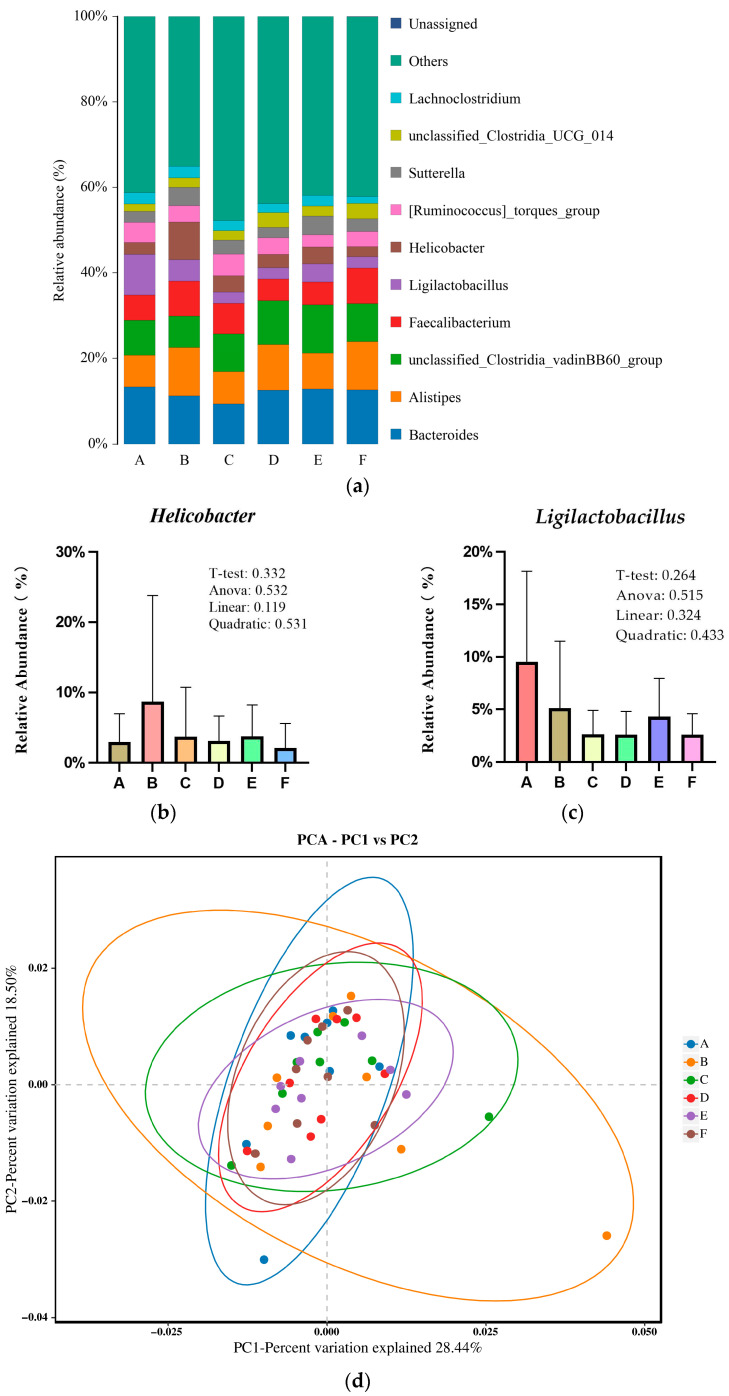
A, B, C, D, E and F are the positive diet, low SBM diet, low SBM with 0.10%, 0.15%, 0.20% and 0.25% raffinose supplementation groups, respectively. (**a**) Relative abundance of bacterial genus in the cecal digesta of broilers. (**b**) Relative abundance of *Helicobacter* at the genus level. All colors represent *Helicobacter*; (**c**) Relative abundance of *Ligilactobacillus* at the genus level. All colors represent *Ligilactobacillus*; The *T*-test is the *p*-value for the comparison of the positive diet versus the low SBM group, while the Anova, linear, and quadratic are the *p*-values for the comparisons among the low SBM and low SBM with 0.10%, 0.15%, 0.20% and 0.25% raffinose supplementation groups in Figure 3b and Figure 3c, respectively. (**d**) PCA of the all groups.

Figure 3a shows the structure of the bacterial community of all groups in the broiler cecal samples at the genus level. The sequences were more abundant in an “others” genus with average relative abundance >40% among groups, and the 10 major classified genera across all groups were the *Bacteroides*, *Alistipes*, *unclassified_Clostridia_vadinBB60_group*, *Faecalibacterium*, *Ligilactobacillus*, *Helicobacter*, *[Ruminococcus]_torques_group*, *Sutterella*, *unclassified_Clostridia_UCG_014*, *Lachnoclostridium*, *others* and *unassigned*, with relative abundance >1%. At the genus level, the abundance of *Helicobacter* (8.68%) was numerically highest in the low SBM group, while *Ligilactobacillus* (9.54%) was most abundant in the positive group (Figure 3b,c). Principal coordinates analysis revealed a separation trend between the positive diet and low SBM groups (Figure 3d).

## 4. Discussion

In the present study, we surprisingly found that a low SBM diet significantly increases the mortality of broilers, but supplementing raffinose to a low SBM diet can reverse this phenomenon. Meanwhile, we further observed that the abundance of Campylobacterota and *Helicobacter* species increased in the cecum of broilers that were fed a low SBM diet, but supplemented raffinose decreased the Campylobacterota and *Helicobacter* abundance. Moreover, a linear trend analysis indicated a potential decrease in Campylobacterota abundance with an increasing raffinose dose (linear *p* = 0.094), which aligns with the observed pattern and may suggest a beneficial modulation of the microbiota. Campylobacteriosis is associated with the consumption of broiler meat and has been the leading reported foodborne gastroenteritis in the European Union (EU) since 2005 [24]. Empirical evidence indicates that a 10- to 1000-fold reduction in Campylobacter levels on poultry carcasses significantly lowers the incidence of human Campylobacteriosis [25]. *Helicobacter* has been linked with enteritis and hepatitis in broiler chickens and laying hens, and diarrhea, gastroenteritis, and liver disease in humans [26], which can be considered a food borne human pathogen. These results indicated that increasing the intestinal bad bacteria is the main reason for the increased mortality of broilers that were fed a low SBM diet.

Meanwhile, supplementing raffinose to a low SBM diet decreases the mortality of broilers, maybe due to raffinose, which was linked to the prevention and inhibition of intestinal pathogenic bacteria colonization and promotion of gut health [27,28,29,30]. A Campylobacter jejuni infection can activate myosin light chain kinase and induce cytoskeleton contraction, leading to the separation of tight junction proteins such as ZO-1 and occludin from the junction, thus damaging the intestinal barrier and causing inflammatory diarrhea [31]. Indeed, the present study observed that dietary supplementation to a low SBM diet with raffinose decreased the Campylobacterota and *Helicobacter* abundance, caused changes in the SCFA profile, and improved the ileal barrier function. Similarly, Yushanaji et al. [17] found that adding raffinose to a yellow-feathered chicken diet balanced their gut bacteria and had a positive effect on their intestinal development and health markers. Additionally, the SCFAs, the end products of microbial carbohydrate fermentation in the gut, serve as biomarkers of intestinal microbiota functionality and ecological balance [32]. Vimon et al. [33] suggested that the SCFA-mediated acidification likely promoted beneficial microbiota proliferation and microbial community restructuring while simultaneously providing energetic substrates for intestinal epithelial development and fortifying tight junction protein mechanisms, collectively contributing to improved gut health. Shehata et al. [34] found that in ovo injection of raffinose positively affected cecal microbial populations, which are intestinal function-related genes. Moreover, submucosal immune cells, stimulated by the gut microbiota or its constituents, release cytokines that activate epithelial cells. This activation, in turn, induces the expression of TJ proteins, which are essential for maintaining the integrity of the intestinal epithelial barrier [35,36]. Similarly, in the present study, the increase in expression of ileal TJ genes may relate to supplementation raffinose to a low SBM diet. Meanwhile, previous studies reported that raffinose can act as an immunomodulator to relieve allergy reactions, elevated the serum IgG and IgA concentration, and elicited a humoral immune response [12,37]. Although the existing studies have not directly pointed out the clear effect of Raffinose on the expression of glucose transporter genes, considering the effect of raffinose as a prebiotic on intestinal morphology and function [14,17], it is speculated that raffinose may indirectly affect the expression of SLC2A2 and SLC5A1 by improving intestinal health, thereby improving glucose absorption. These results suggest that the main reason why adding raffinose to a low SBM diet can reduce the mortality rate of broilers may be that raffinose enhances intestinal barrier function by regulating gut microbiota and activating the immune system.

Activation of the immune system can lead to nutrient redistribution, ultimately resulting in decreased production performance. Consistently, in the current study, graded raffinose inclusion in a low SBM diet presented dose-dependent modulations: ADG and ADFI during 22 to 42 d, GE and DM availability, and the expression of duodenal SLC5A1 gene exhibited a linear decrease, while the expression of the jejunal SLC5A1 gene demonstrated a linear increase. In previous studies, when raffinose entered the intestines of animals without digestion, it was fermented by intestinal microorganisms, resulting in flatulence, discomfort and even diarrhea. This discomfort may indirectly affect the intake of food and the absorption of other nutrients, thereby reducing the overall utilization of nutrients [38]. Zeng et al. [12] found that the growth performance and the ATTD (apparent total tract digestibility) of CP, CF, DM and GE was decreased in the 0.5% raffinose supplementation groups and further verified that the decrease in nutrient digestibility was induced by raffinose instead of the lessened feed intake in piglets. Based on the results of the current study and these previous studies, we believe that optimum raffinose supplementation to a low SBM diet can balance the immune response and growth performance of broilers. Based on the raffinose content in dry soybean seeds (approximately 12.6 mg/kg), a 10-percentage-point reduction in dietary soybean meal inclusion would decrease the dietary raffinose concentration by about 1.26 mg/kg of diet. Therefore, when using low SBM diet application in broiler diets, we need to consider the content of functional soybean oligosaccharide.

We believe that the decrease in nutrient digestibility, changes in microbiota composition, and even the increase in mortality observed in the low SBM group may be partly due to differences in nutrient release kinetics, not just the decrease in the soybean meal effect. A low SBM diet containing different levels of crystalline amino acids (lysine, methionine, threonine, tryptophan) may represent a key nutritional confounding factor. Studies have shown that broiler chickens that were fed low-protein diets supplemented with various combinations of crystalline amino acids exhibited effects in the growth performance, nutrient utilization, and digestive dynamics of protein and amino acids [39].

Raffinose can enhance the intestinal barrier function and healthy microbial community, improve the resistance of broilers to pathogens and reduce the incidence of intestinal diseases, which means that the cost of veterinary drugs in disease prevention and treatment will be reduced. Future studies can clarify how enhanced intestinal barrier function promotes nutrient absorption, which will provide a more comprehensive mechanism explanation.

## 5. Conclusions

Collectively, we found that reducing SBM in diets by 10% increased the mortality and the cecal foodborne bacterial, such as Campylobacterota and *Helicobacter* abundance in white-feather broilers. Although adding raffinose to a low SBM diet had no marked negative impact on the growth performance, raffinose can modulate the cecal microbiota structure, reduce Campylobacterota and *Helicobacter* abundance, increase SCFA production, and improve ileal barrier function to elevate the survival rate of broilers. Based on the dose–response effects observed, an optimum raffinose supplementation level of 0.25% is recommended for low SBM broiler diets. These results suggested that when using low SBM diet application in broiler diets, we need to consider the content of functional soybean oligosaccharide, which provides new insight into reducing soybean substitution in the poultry feed industry.

## Figures and Tables

**Table 1:** Composition and nutrient levels of the positive and low SBM diets (on fed-basis), %.

Ingredients	1–21 d		22–42 d	
	Positive Diet	Low SBM Diet	Positive Diet	Low SBM Diet
Corn	51.13	51.04	55.75	56.87
Soybean meal	36.00	26.00	30.00	20.00
Corn gluten meal	1.60	4.95	3.03	6.50
Rapeseed meal	0.50	2.95	0.50	4.50
DDGS	0.50	4.95	0.50	2.20
Cottonseed meal	0.96	0.96	0.96	0.96
Soybean oil	4.64	4.29	5.11	4.64
Dicalcium phosphate	1.99	1.85	1.68	1.66
Calcium carbonate	1.25	1.35	1.20	1.20
Sodium chloride	0.30	0.30	0.30	0.30
Choline chloride (50%)	0.15	0.15	0.15	0.15
Vitamin premix ^1^	0.03	0.03	0.03	0.03
Mineral premix ^2^	0.20	0.20	0.20	0.20
L-lysine	0.17	0.38	0.14	0.34
DL-methionine	0.19	0.16	0.10	0.07
L-threonine	0.12	0.15	0.08	0.10
L-tryptophan	0.00	0.02	0.00	0.01
Rice bran	0.27	0.27	0.27	0.27
Total	100.00	100.00	100.00	100.00
Calculated nutrient levels (%)				
ME (MJ/kg)	12.54	12.54	12.96	12.96
Crude protein	21.50	21.50	20.00	20.00
Calcium	1.00	1.00	0.90	0.90
Non-phytate phosphorus	0.45	0.45	0.40	0.40
Digestible lysine	1.15	1.15	1.00	1.00
Digestible methionine	0.50	0.50	0.40	0.40
Digestible threonine	0.81	0.81	0.72	0.72
Digestible tryptophan	0.21	0.21	0.18	0.18

^1^ Vitamin premix provides the following per kg of final diet: vitamin A, 8000 IU; vitamin D3, 2000 IU; vitamin E, 20 IU; vitamin K3, 0.5 mg; vitamin B1, 2.0 mg; vitamin B2, 8.0 mg; vitamin B6, 3.5 mg; vitamin B12, 0.01 mg; pantothenic acid, 10.0 mg; niacin, 35.0 mg; folic acid, 0.55 mg; and biotin, 0.18 mg. ^2^ Mineral premix provides the following per kg of final diet: copper (CuSO_4_·5H_2_O), 8.0 mg; iron (FeSO_4_·H_2_O), 80.00 mg; zinc (ZnSO_4_·H_2_O), 80.00 mg; manganese (MnSO_4_·H_2_O), 100.00 mg; iodine (KI), 0.70 mg; and selenium (Na_2_SeO_3_), 0.30 mg.

**Table 2:** Design sequence of target genes and reference primers ^1^.

Gene Name	Orientation	Primer Sequence (5′-3′)	Accession Number	Product Size/bp	Amplification Efficiency
β-actin	Forward	AATCAAGATCATTGCCCCACCT	NM_205518.2	173	≥95%
	Reverse	TGGGTGTTGGTAACAGTCCG			
OCLN	Forward	ACGGCAGCACCTACCTCAA	NM_205128.1	123	≥95%
	Reverse	GGGCGAAGAAGCAGATGAG			
TJP1	Forward	TTCAGGTGTTTCTCTTCCTCCTC	XM_040680628.2	130	≥95%
	Reverse	CTGTGGTTTCATGGCTGGATC			
CLDN1	Forward	GTGTGTTTGTTGCTGTGA	NM_001013611.2	151	≥95%
	Reverse	ACTCTGTTGCCATACCAT			
CLDN3	Forward	GTCATCTTCCTGCTCTCC	NM_204202.2	86	≥95%
	Reverse	AGCGGGTTGTAGAAATCC			
SCL2A1	Forward	TGTCCTGCTGGTCACACTTC	NM_207178.2	88	≥95%
	Reverse	CGTGACTATGAGCTGGCGAT			
SLC5A1	Forward	TGCAGTTAATACAGCGGGAGA	NM_001397792.1	87	≥95%
	Reverse	TGTGGACACCAAATGCTTCA			
MUC2	Forward	GGTGAGTCATGGTGGCTGTGTAAC	XM_040673070.2	112	≥95%
	Reverse	CATTGGAGCAGGTGGGTTTAGGAG			

^1^ β-actin: actin, beta; OCLN: occluding; TJP1: Tight junction protein 1, CLDN1: Claudin 1; CLDN3: Claudin 3; SCL2A1: Solute carrier family 2 member 2; SCL5A1: Solute carrier family 5 member 1; and MUC2: Mucin 2.

**Table 3:** Effects of low SBM diet and raffinose supplementation on productive performance of broilers ^1^.

Items ^2^	Normal	Low Soybean Meal Diets with Raffinose Supplementation, %					SEM	*p*-Value		
	Diet	0.00	0.10	0.15	0.20	0.25		Anova	Linear	Quadratic
1–21 d										
1 d BW, g	46.90	46.60	46.70	46.60	46.60	46.80	0.19	0.972	0.805	0.798
21 d BW, g	977.1	947.6	935.3	930.0	950.7	932.4	18.49	0.904	0.744	0.786
ADG, g/day	44.30	42.90	42.32	42.07	43.06	42.17	0.88	0.902	0.743	0.788
ADFI, g/day	61.90	60.80	60.10	59.05	63.69	60.56	1.15	0.081	0.497	0.554
F:G	1.40	1.43	1.43	1.40	1.46	1.41	0.02	0.366	0.884	0.994
Mortality, %	1.25	5.00 ^a^	1.25 ^ab^	1.25 ^ab^	5.00 ^a^	0.00 ^b^	1.40	0.049	0.122	0.624
22–42 d										
42 BW, g	2737	2874	2735	2790	2787	2804	73.33	0.763	0.563	0.313
ADG, g/day	83.80	90.20	85.80	87.90	85.60	89.10	3.06	0.783	0.678	0.324
ADFI, g/day	156.7	170.9 *	160.8	165.3	167.2	167.9	4.43	0.596	0.804	0.180
F:G	1.87	1.90	1.87	1.89	1.96	1.89	0.03	0.214	0.593	0.811
Mortality, %	5.73	11.51	6.94	8.33	3.17	8.33	2.85	0.228	0.088	0.200
1–42 d										
ADG, g/day	65.00	67.30	65.10	66.60	65.20	65.60	1.91	0.908	0.543	0.719
ADFI, g/day	108.4	114.8	109.5	111.4	114.5	112.9	2.50	0.535	0.930	0.232
F:G	1.67	1.71	1.68	1.68	1.76	1.72	0.03	0.227	0.377	0.349
Mortality, %	6.25	14.29 *	7.50	8.75	7.14	7.50	2.56	0.348	0.190	0.382
EPI	365.6	334.8	356.9	350.7	329.7	359.0	18.80	0.654	0.616	0.831

* Indicates a significant difference between the positive and low SBM diets, as determined by Student’s *t*-test (*p* < 0.05). ^a, b^ Means within a row with different superscripts are different at *p* < 0.05. ^1^ Values are the mean of 8 replicates of 10 chickens each. ^2^ BW: body weight; ADG: average daily gain; ADFI: average daily feed intake; and F:G: feed to gain ratio.

**Table 4:** The effects of low SBM diet and raffinose supplementation on dietary nutrient utilization of broilers ^1^, %.

Items ^2^	Normal	Low Soybean Meal Diets with Raffinose Supplementation, %					SEM	*p*-Value		
	Diet	0.00	0.10	0.15	0.20	0.25		Anova	Linear	Quadratic
GE	79.83	81.12	81.07	81.74	80.26	76.10	1.48	0.072	0.043	0.053
CP	57.78	60.12	61.67	61.75	59.26	50.37	3.36	0.118	0.079	0.050
EE	90.51 *	87.22	87.77	88.57	89.14	85.43	1.45	0.433	0.746	0.156
DM	75.95	78.29 ^ab^	77.01 ^ab^	78.73 ^a^	76.45 ^ab^	70.33 ^b^	2.00	0.036	0.020	0.063
Ash	47.55	49.24	39.44	34.22	46.63	37.26	4.18	0.077	0.134	0.225
Ca	63.21	58.58	60.44	62.85	62.90	53.87	3.76	0.426	0.689	0.122
TP	50.36	50.53	55.49	55.21	51.06	41.43	4.07	0.123	0.157	0.024

* Indicates a significant difference between the positive and low SBM diets, as determined by Student’s *t*-test (*p* < 0.05). ^a, b^ Means within a row with different superscripts are different at *p* < 0.05. ^1^ Values are the mean of 8 replicates of 10 chickens each. ^2^ GE: gross energy; CP: crude protein; EE: ether extract; DM: dry matter; Ash: crude ash; Ca: calcium; and TP: total phosphorus.

**Table 5:** The effect of low SBM diet and raffinose supplementation on the nutrient transport-related gene expression in duodenum and jejunum of broilers ^1^.

Items ^2^		Normal	Low Soybean Meal Diets with Raffinose Supplementation, %					SEM	*p*-Value		
		Diet	0.00	0.10	0.15	0.20	0.25		Anova	Linear	Quadratic
Duodenum											
21 d	SLC2A2	1.00	0.67	1.57	0.47	1.59	1.07	0.40	0.089	0.447	0.629
	SLC5A1	1.00	0.91	0.90	1.26	1.19	1.00	0.20	0.356	0.446	0.505
42 d	SLC2A2	1.00	0.77	1.14	0.55	0.35	0.44	0.24	0.101	0.733	0.357
	SLC5A1	1.00	1.06 ^a^	0.91 ^ab^	0.43 ^b^	0.49 ^b^	0.56 ^ab^	0.17	0.046	0.011	0.394
Jejunum											
21 d	SLC2A2	1.00	0.73	1.00	0.16	0.89	0.87	0.30	0.258	0.980	0.772
	SLC5A1	1.00	0.91	0.99	0.99	1.33	0.84	0.22	0.687	0.968	0.475
42 d	SLC2A2	1.00	2.03	2.71	1.46	1.84	3.54	0.62	0.182	0.420	0.315
	SLC5A1	1.00	0.95	1.31	1.52	2.14	1.89	0.33	0.220	0.023	0.833

^a, b^ Means within a row with different superscripts are different at *p* < 0.05. ^1^ Values are the mean of 8 replicates of 10 chickens each. ^2^ SLC2A2: solute carrier family 2 member 2, and SLC5A1: solute carrier family 5 member 1.

**Table 6:** The effect of a low SBM diet and raffinose supplementation on the barrier-related gene expression in the ileal mucosa of broilers ^1^.

Items ^2^		Normal	Low Soybean Meal Diets with Raffinose Supplementation, %					SEM	*p*-Value		
		Diet	0.00	0.10	0.15	0.20	0.25		Anova	Linear	Quadratic
21 d	OCLN	1.00	0.74	0.76	0.58	0.52	0.79	0.11	0.241	0.607	0.267
	TJP1	1.00	0.73	0.65	0.78	0.60	0.71	0.12	0.834	0.739	0.896
	CLDN1	1.00	0.76	1.01	0.73	0.83	0.89	0.20	0.871	0.830	0.844
	CLDN3	1.00	0.68	0.86	0.56	0.52	0.88	0.12	0.126	0.864	0.419
	MUC2	1.00	1.04	0.76	0.65	0.45	0.75	0.19	0.325	0.120	0.267
42 d	OCLN	1.00	1.09	1.05	1.22	0.63	0.90	0.19	0.337	0.260	0.641
	TJP1	1.00	1.05	1.30	1.48	1.03	1.00	0.19	0.365	0.746	0.093
	CLDN1	1.00	0.80	1.46	1.15	0.99	0.81	0.17	0.101	0.778	0.012
	CLDN3	1.00	0.74	0.94	0.91	0.75	0.81	0.18	0.903	0.954	0.562
	MUC2	1.00	1.02	0.89	1.05	0.56	0.69	0.20	0.405	0.156	0.703

^1^ Values are the mean of 8 replicates of 10 chickens each. ^2^ OCLN: occludin, TJP1: Tight junction protein 1, CLDN1: Claudin 1, CLDN3: Claudin 3, and MUC2: Mucin 2.

**Table 7:** Effects of low SBM diet and raffinose supplementation on cecal short-chain fatty acid content of broilers, %.

Items	Normal	Low Soybean Meal Diets with Raffinose Supplementation, %					SEM	*p*-Value		
	Diet	0.00	0.10	0.15	0.20	0.25		Anova	Linear	Quadratic
Acetic acid	12.81	11.77	10.62	11.89	12.38	13.63	1.45	0.692	0.328	0.332
Propionic acid	4.73	3.50	3.93	4.53	3.68	4.31	0.58	0.729	0.434	0.677
Isobutyric acid	1.49	1.18 ^b^	1.01 ^b^	1.19 ^b^	3.38 ^a^	6.97 ^a^	0.52	<0.001	<0.001	<0.001
Butyric acid	4.87	6.25	4.81	5.58	6.18	6.97	0.71	0.296	0.383	0.062
Isovaleric acid	4.61	4.05	3.49	3.65	3.47	3.26	0.31	0.530	0.112	0.811
Valeric acid	0.85	1.11	0.96	1.12	1.16	1.17	0.15	0.875	0.626	0.554
Caproic	23.85	23.95	25.11	19.32	23.23	19.69	2.27	0.294	0.194	0.759

^a, b^ Means within a row with different superscripts are different at *p* < 0.05.

## Data Availability

Data are available upon request from the corresponding author.
